# Whole-Exome Sequencing Analysis of Idiopathic Hypogonadotropic Hypogonadism: Comparison of Varicocele and Nonobstructive Azoospermia

**DOI:** 10.1007/s43032-023-01337-2

**Published:** 2023-09-07

**Authors:** Ziyang Ma, Yi Dai, Lei Jin, Yi Luo, Chen Guo, Rui Qu, Shengyin He, Yugao Liu, Yu Xia, Huan Liu, Lingnan Kong, Miaomiao Xu, Lanlan Zhang, Yue Zhao, Yushanjiang Suliya, Dongzhi Yuan, Luo Yang

**Affiliations:** 1 Department of Physiology, West China College of Basic Medicine and Forensic Medicine, Sichuan University, Chengdu, Sichuan China; 2 Urology/Pelvic Floor Surgery, West China Fourth Hospital, Sichuan University, Chengdu, Sichuan China; 3 West China School of Public Health, Sichuan University, Chengdu, Sichuan China; 4 Sichuan University, Chengdu, Sichuan China; 5 Department of Laboratory Sciences of Public Health, West China School of Public Health and West China Fourth Hospital, Sichuan University, Chengdu, Sichuan China

**Keywords:** Normosmic idiopathic hypogonadotropic hypogonadism, nIHH, Whole exon sequence, Varicocele

## Abstract

As a rare disease leading to male infertility, idiopathic hypogonadotropic hypogonadism (IHH) has strong heterogeneity of clinical phenotype and gene mutation. At present, there is no effective diagnosis and treatment method for this disease. This study is to explore the possible new pathogenic gene of idiopathic hypogonadotrophic hypogonadism and the pathological mechanism affecting its occurrence. We performed a whole-exome sequencing on 9 patients with normosmic idiopathic hypogonadotropic hypogonadism (nIHH), 19 varicocele patients with asthenospermia, oligospermia, or azoospermia, 5 patients with simple nonobstructive azoospermia, and 13 normal healthy adult males and carried out comparative analysis, channel analysis, etc. After preliminary sequencing screening, 309–431 genes harbouring variants, including SNPs and indels, were predicted to be harmful per single patient in each group. In genetic variations of nIHH patients’ analysis, variants were detected in 10 loci and nine genes in nine patients. And in co-analysis of the three patient groups, nine nIHH patients, 19 VC patients, and five SN patients shared 116 variants, with 28 variant-harbouring genes detected in five or more patients. We found that the NEFH, CCDC177, and PCLO genes and the Gene Ontology pathways GO:0051301: cell division and GO:0090066: regulation of anatomical structure size may be key factors in the pathogenic mechanism of IHH. Our results suggest that the pathogenic mechanism of IHH is not limited to the central nervous system effects of GnRH but may involve other heterogeneous pathogenic genetic variants that affect peripheral organs.

**Supplementary Information:**

The online version contains supplementary material available at 10.1007/s43032-023-01337-2.

## Introduction

Idiopathic hypogonadotropic hypogonadism (IHH) is a rare disease with clinical manifestations of azoospermia, hypogonadotropism, pubertal retardation or deletion, and infertility. There is a significant difference in incidence between men (approximately 1:30,000) and women (1:125,000) [1, 2]. It is currently thought that insufficient GnRH secretion is the main cause of IHH. This may be due to abnormal GnRH neuron development, differentiation, or activation; failure of GnRH activation; or insufficient GnRH secretion [1, 3].

A type of IHH known as Kallman syndrome (KS) is related to the development of GnRH neurons [1, 3]. Patients with KS show developmental disorders of the reproductive and olfactory systems [4]. Unlike KS, normosmic idiopathic hypogonadotropic hypogonadism (nIHH) shows complex clinical phenotypic diversity, and its pathogenic mechanism cannot be explained. To investigate the pathogenesis of IHH, many whole-genome sequencing analyses have been performed, resulting in the identification of more than 60 genes associated with IHH [5, 6]. More than 40 of these genes have been shown to affect the development of the reproductive system in animal experiments [7].However, clinical data do not support the hypothesis that IHH stems from the pathogenesis of GnRH neurons [1]. First, despite hypogonadotropism in IHH patients, hormone supplementation is not an effective treatment, and more than half of patients fail to regain fertility. Second, there is extensive genetic heterogeneity among IHH patients, and large-scale next-generation sequencing of IHH patients has shown that no single genetic variant or class of genetic variants is widely present in IHH patients. Variants in the most frequently involved gene, *FGFR1*, are present in no more than 10% of IHH patients. Although variants in more than 60 genes have been found to be associated with the development of IHH, these genetic data are not sufficient to suggest causation. In addition, most IHH patients have other symptoms, such as hearing impairment, affective disorder, or limb coordination disorder, and the pathogenic mechanism in these patients cannot be explained by the GnRH neuronal origin hypothesis. Moreover, like patients with simple nonobstructive azoospermia (SN, NOA with azoospermia phenotype only), patients with nIHH have azoospermia, but they do not have nervous system abnormalities. Many pathogenic mechanisms of diseases such as SN do not involve the hypothalamus or pituitary gland but are characterised by gonadal dysfunction, such as abnormalities of the testes (including abnormal spermatogenesis and Sertoli cells). Although these abnormalities are heterogeneous, hormone therapy is ineffective in some IHH patients, which may lead to common non-central abnormalities that cause nIHH and SN.

Next-generation sequencing technology has facilitated the detection of genetic variants on a large scale, but studies targeting diseases with genetic heterogeneity remain difficult. In case–control studies, the frequencies of variants in many genes have varied greatly in different populations studied, due to factors such as the characteristics of the population, ethnicity, and geographical location, making it difficult to identify the exact genes associated with the disease. Taking the normal population as the control, due to differences in the natural variation rate of many genes in different populations, the prediction of pathogenic genes associated with rare diseases, such as IHH, is often inaccurate (false positives). For monogenic rare diseases, the detection of the causative gene is largely obscured by the population variation rate. Traditionally, case–control studies of IHH have been conducted to screen for variants by next-generation sequencing, which to some extent increases the likelihood that IHH will be defined and interpreted as a single disease. However, it is also possible that the genetic pathology underlying IHH may be shared with other NOA. Under that premise, overlapping pathologies may be overemphasised, and the mutations only associated with IHH may be ignored. *CHD7* is one of the pathogenic genes associated with coloboma, heart defects, atresia choanae (CHARGE) syndrome, and IHH, while reproductive system dysplasia, the main clinical manifestation of IHH, is only one of the secondary symptoms of CHARGE syndrome [8, 9]. Studies have shown that variation in the *PORKR2* gene is markedly higher in the Maghrib population (23.3%) than in European populations (5.1%), and it has more reference significance for the diagnosis of KS [10].

In this study, we focused on the Han population in Southwest China. In addition to nIHH patients and normal males, varicocele patients with asthenospermia, oligospermia, or azoospermia (VC) and simple nonobstructive azoospermia (SN) (the patient only has a phenotype with sperm count of 0) were included in the study as 2 other types of NOA (here, we include a wide range of nonobstructive sperm with low quantity or abnormal quality in NOA) to investigate the broader genetic pathology of nIHH. Exome sequencing analysis was combined with pathway analysis to investigate the independent genetic pathogenic basis of nIHH.

## Materials and Methods

### Subjects and Clinical Evaluation

We have included 9 patients with normosmic idiopathic hypogonadotropic hypogonadism (nIHH); 19 varicocele patients with asthenospermia, oligospermia, or azoospermia; 5 patients with simple nonobstructive azoospermia; and 13 normal healthy adult males.

The research included nine male nIHH patients (Table 1). nIHH patients were included in the study if they had (a) low concentrations of follicle-stimulating hormone (FSH) and luteinising hormone (LH); (b) a low serum testosterone concentration; (c) a zero sperm count; (d) retardation or absence of puberty; (e) computed tomography and magnetic resonance imaging results showing no obvious organic injury in the pituitary region; and (f) a normal chromosome karyotype [1].

**Table 1:** Patient information

Patient	Age	Diagnosis	Main symptoms	Accompanying symptoms	Sperm	LH	T
nIHH1	26	nIHH	Cryptorchidism, micropenis	Renal cyst, obesity	Azoospermia	0.2	0.03
nIHH2	21	nIHH	Cryptorchidism, micropenis	-	Azoospermia	< 0.1	< 0.01
nIHH3	23	nIHH	Cryptorchidism, micropenis	-	Azoospermia	0.25	0.02
nIHH4	20	nIHH	Cryptorchidism, micropenis	Varicocele, absence of right kidney	Azoospermia	0.23	0.03
nIHH5	25	nIHH	Cryptorchidism, micropenis	-	Oligozoospermia	< 0.1	< 0.01
nIHH6	24	nIHH	Cryptorchidism, micropenis	Varicocele, obesity	Azoospermia	< 0.1	< 0.01
nIHH7	24	nIHH	Cryptorchidism, micropenis	Scleroderma of head	Azoospermia	0.16	< 0.01
nIHH8	28	nIHH	Cryptorchidism, micropenis	Bone tumour and osteomyelitis	Azoospermia	0.17	< 0.01
nIHH9	24	nIHH	Cryptorchidism, micropenis	Obesity	Azoospermia	< 0.1	< 0.01
SN1	28	SN	Nonobstructive azoospermia	-	Azoospermia	-	4.19
SN2	37	SN	Nonobstructive azoospermia		Azoospermia	12.2	5.08
SN3	34	SN	Nonobstructive azoospermia	-	Azoospermia	-	-
SN4	24	SN	Nonobstructive azoospermia		Azoospermia	-	5.27
SN5	36	SN	Nonobstructive azoospermia		Azoospermia	-	-
VC1	26	VC	Bilateral varicocele	-	Oligozoospermia	-	-
VC2	33	VC	Left varicocele	-	Azoospermia	-	-
VC3	29	VC	Bilateral varicocele	-	Asthenospermia	-	-
VC4	16	VC	Bilateral varicocele	-	Oligozoospermia	-	-
VC5	23	VC	Left varicocele	-	Oligozoospermia	-	-
VC6	18	VC	Left varicocele	-	Azoospermia	-	-
VC7	28	VC	Bilateral varicocele	-	Azoospermia	-	-
VC8	37	VC	Left varicocele	-	Oligozoospermia	-	-
VC9	21	VC	Bilateral varicocele	-	Azoospermia	-	-
VC10	41	VC	Left varicocele	-	Asthenospermia	-	-
VC11	19	VC	Bilateral varicocele	-	Azoospermia	-	-
VC12	25	VC	Left varicocele	-	Asthenospermia	-	-
VC13	29	VC	Bilateral varicocele	-	Oligozoospermia	-	-
VC14	29	VC	Bilateral varicocele	-	Oligozoospermia	-	-
VC15	27	VC	Bilateral varicocele	-	Asthenospermia	-	-
VC16	39	VC	Bilateral varicocele	-	Azoospermia	-	-
VC17	24	VC	Left varicocele	-	Asthenospermia	-	-
VC18	20	VC	Left varicocele	-	Asthenospermia	-	-
VC19	29	VC	Bilateral varicocele	-	Asthenospermia	-	-

We included five SN patients and 19 VC patients. SN patients were included if they had the following: (a) sperm count is 0; (b) normal FSH and LH concentrations; (c) no obvious organic damage to the hypothalamus or pituitary gland; (d) a normal chromosome karyotype; (e) normal reproductive tract and urethral structure; (f) no obvious organic damage to the gonads; and (g) no other type of azoospermia [11]. Varicocele patients with asthenospermia, oligospermia, or azoospermia were included if they had the following: (a) a unilateral or bilateral varicocele under colour Doppler ultrasound and (b) sperm count is 0 or less than or equal to 20*10^6^/ml, or progressive sperm motility are less than 60% [12].

For the normal male control (NC) group, we selected 13 healthy young men in Southwest China. The young men were eligible for the NC group if they (a) were aged 21 to 26; (b) had a body mass index of 20–25; (c) had normal sperm morphology under microscopic examination; (d) had a sperm count > 20 × 10^6^/mL; (e) had a sperm motility rate > 80%; (f) had normal sperm motility; (g) had no hereditary disease affecting their growth and development or their reproductive system; and (h) had no serious disease that may affect the results of the study. All patients were diagnosed and treated at the West China Fourth Hospital, Chengdu, China. After being informed of the details and specific risks of the study, they voluntarily joined the study.

### Analysis Methods and Strategy

#### Sequencing and Data Analysis

##### DNA Extract and Detect

Genomic DNA extracted from peripheral blood for each sample was fragmented to an average size of 180 ~ 280 bp and subjected to DNA library creation using established Illumina paired-end protocols. The Agilent SureSelect Human All ExonV6 Kit (Agilent Technologies, Santa Clara, CA, USA) was used for exome capture according to the manufacturer’s instructions. The Illumina Novaseq 6000 platform (Illumina Inc., San Diego, CA, USA) was utilized for genomic DNA sequencing in Novogene Bioinformatics Technology Co., Ltd (Beijing, China) to generate 150-bp paired-end reads with a minimum coverage of 10 × for ~ 99% of the genome (mean coverage of 100 ×).

##### Data Analysis

After sequencing, basecall files conversion and demultiplexing were performed with bcl2fastq software (Illumina). The resulting fastq data were submitted to in-house quality control software for removing low quality reads and then were aligned to the reference human genome (hs37d5) using the Burrows-Wheeler Aligner (bwa)^13^, and duplicate reads were marked using sambamba tools [14].

##### SNP/INDEL calling

Single nucleotide variants (SNVs) and indels were called with samtools to generate gVCF [15]. The raw calls of SNVs and INDELs were further filtered with the following inclusion thresholds: (1) read depth > 4; (2) root-mean-square mapping quality of covering reads > 30; and (3) the variant quality score > 20.

##### CNV calling

The copy number variants (CNVs) were detected with software CoNIFER (V0.2.2) [16].

##### Annotation

Annotation was performed using ANNOVAR (2017June8) [17]. Annotations included minor allele frequencies from public control data sets as well as deleteriousness and conservation scores enabling further filtering and assessment of the likely pathogenicity of variants.

#### Rare Variant Filtering

Filtering of rare variants was performed as follows: (1) variants with a MAF less than 0.01 in 1000 genomic data (1000g_all) [18], esp6500siv2_all, gnomAD data (gnomAD_ALL and gnomAD_EAS), and in-house Novo-Zhonghua exome database from Novogene; (2) only SNVs occurring in exons or splice sites (splicing junction 10 bp) are further analysed since we are interested in amino acid changes. (3) Then, synonymous SNVs which are not relevant to the amino acid alternation predicted by dbscSNV are discarded; the small fragment non-frameshift (< 10 bp) indel in the repeat region defined by RepeatMasker is discarded. (4) Variations are screened according to scores of SIFT [19], Polyphen [20], MutationTaster [21], and CADD [22] software. The potentially deleterious variations are reserved if the score of more than half of these four software support harmfulness of variations [23]. Sites (> 2 bp) that did not affect alternative splicing were removed.

#### ACMG Gene Screening and Comparison

In 2015, the ACMG developed standards and guidelines for the interpretation of sequence variants. These standards have become the gold standard for data interpretation after high-throughput sequencing [24]. The variant classification system developed by the ACMG recommends the use of specific standard terms. The system uses the classifications pathogenic, likely pathogenic, uncertain significance, likely benign, or benign to describe variants found in pathogenic genes of Mendelian diseases.

We performed ACMG analysis on the variant-harbouring genes of all patients after preliminary screening, i.e. next-generation sequencing, quality inspection, coverage determination, and in-depth screening, and identified the variants classified as pathogenic and likely pathogenic. The results of the ACMG analysis for the nIHH group were compared with previously identified pathogenic genes of nIHH.

#### Metascape Pathway and Pathway Enrichment Analysis

**Fig. 1:**
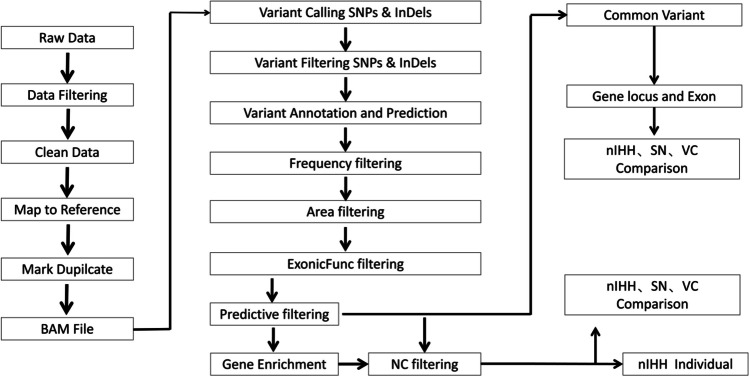
Sequencing and analysis process. Predictive filtering generated from the raw data obtained from the second generation sequencing after routine quality control, basic screening, and hazard prediction. On the one hand, it generates mutation gene analysis and pathway analysis of nIHH, SN, and VC groups after removing NC data; on the other hand, four groups of different mutation site analysis of the same gene were generated

Metascape (metascape.org) is a genome-wide data analysis network platform that integrates more than 40 omics data analyses. The platform provides a series of analysis modes and strategies, including gene pathway and process enrichment analysis, including Kyoto Encyclopedia of Genes and Genomes (KEGG) pathway and Gene Ontology (GO) biochemical pathway analyses; protein–protein interaction analysis, including string6, biogrid7, and omnipath8; and DisGeNET associated disease analysis. The results are presented in easy-to-understand data tables and images (Fig. 1).

Our data were processed using the pathway enrichment analysis method provided by Metascape. The analysis included KEGG pathways; GO biological processes; reaction group gene sets; standard pathways; and data from the CORUM, Rust, DisGeNET, PaGenBase, transcription factor targets, WikiPathways, and PANTHER Pathway. Enrichment was based on a *P* value < 0.005, a minimum number of three genes in the same pathway, and an enrichment factor > 1.5. Enrichment values were arranged from small to large. Pathway enrichment was performed for each patient.

#### Screening Strategy

##### Analysis of Mutant Genes and Their Biological Functions

First, we eliminated all genetic variants in the three patient groups that were also detected in the NC group. We also compared the data from the three patient groups after preliminary screening. We analysed the data from the nIHH group, after excluding data from the NC group. Previous studies have identified 63 mutated genes affecting the development of nIHH. We compared the nIHH group data with these previously identified genes and then analysed the variants in the nIHH group based on the ACMG criteria. Finally, we filtered the data for genetic variants that were only present in the nIHH group.

We then used a similar method to screen the genetic variants in both the nIHH and NC groups for pathway enrichment analysis and excluded the enriched pathways that appeared in the NC group from the enriched pathways in the nIHH group. Finally, we obtained the screened enriched pathways for nIHH.

##### Analysis of Common Gene Mutations in nIHH, VC, and SN and Their Biological Functions

After removing the genetic variants identified in the NC group, we compared and screened independent data from the nIHH, VC, and SN groups. We focused on variants that were common to the three groups and analysed those related to spermatogenesis or those with a high level of consistency or large differences between the three groups. Next, we enriched the genes in each group and excluded the enrichment pathway data from the NC group. We combined the enrichment pathway data for the nIHH, VC, and SN groups and identified the enrichment pathways that appeared in all three groups.

We then analysed the data for all four groups without any data excluded. We first counted the common genetic variants present in the four groups, arranged in descending order by the number of co-variants. Of the genes harbouring variants, we selected 18 that most commonly harboured variants and counted their specific variant sites or the exons harbouring the variants (assembly: GRCh37.p13 GCF_000001405.25) to show the distribution of variants located in specific genes in each of the four groups.

## Results

### Preliminary Screening of Genetic Variants in Each Group

**Fig. 2:**
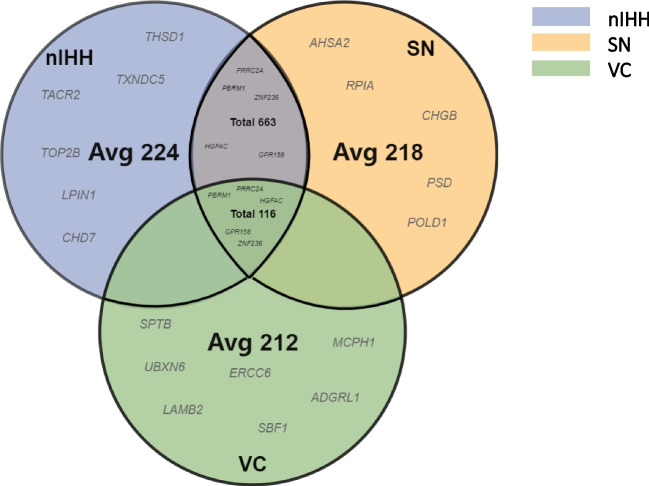
Intra and inter group genes. Avg: after removing NC data, the average number of gene mutations contained in each patient in the group. Each colour circle represents the mutation of its group after removing the NC group data. The intersection of two circles is the mutation gene shared by the two groups, and the intersection of three circles is the mutation gene shared by the three groups

After preliminary sequencing screening, 309–431 genes harbouring variants, including SNPs and indels, were predicted to be harmful per single patient in each group. After eliminating the variants detected in the NC group and comparing the three remaining groups, the number of genes identified per single patient in the IHH, VC, and SN groups was approximately 98–135. Six hundred and forty-five genetic variants were shared between the nIHH and VC groups, and 116 genetic variants were shared by the nIHH, VC, and SN groups (Fig. 2). All analyzed gene mutations occur in the exon region or 2–3 bp away from the exon region and can cause changes in the amino acid sequence of the gene expression protein and are predicted to affect the normal expression of the gene (Supplementary materials).

### Genetic Variants in nIHH Patients

We compared the genetic variants in the nIHH group, after excluding those identified in the NC group, with previously identified IHH pathogenic variants (Tables 2 and 3). Variants were detected in 10 loci and nine genes in nine patients. *CHD7* gene variants were identified in patients nIHH2 and nIHH4. Variants in the remaining genes, *ANOS1*, *FGFR1*, *SEMA7A*, *OTUD4*, *AMH*, *KLB*, *GATA2*, and *POLR3B*, were all detected once.

**Table 2:** Common mutated genes in group nIHH

	Num	nIHH1	nIHH2	nIHH3	nIHH4	nIHH5	nIHH6	nIHH7	nIHH8	nIHH9
PRRC2A	4			PRRC2A			PRRC2A	PRRC2A		PRRC2A
WNK2	3							WNK2	WNK2	WNK2
UTP20	3	UTP20		UTP20	UTP20					
THSD1	3			THSD1		THSD1	THSD1			
SMPD1	3			SMPD1		SMPD1	SMPD1			
SLC16A3	3			SLC16A3				SLC16A3	SLC16A3	
PIK3C2B	3		PIK3C2B			PIK3C2B				PIK3C2B
PBRM1	3	PBRM1						PBRM1	PBRM1	
KRT13	3					KRT13	KRT13		KRT13	
KPNA2	3		KPNA2		KPNA2				KPNA2	
KDM2B	3			KDM2B				KDM2B	KDM2B	
HGFAC	3					HGFAC	HGFAC	HGFAC		
GPR158	3	GPR158		GPR158			GPR158			
CYP2C8	3		CYP2C8	CYP2C8	CYP2C8					
CYB5D2	3		CYB5D2			CYB5D2		CYB5D2		
COL12A1	3		COL12A1	COL12A1				COL12A1		
COL11A2	3	COL11A2	COL11A2		COL11A2					
BICC1	3		BICC1		BICC1					BICC1
ANK1	3		ANK1				ANK1	ANK1		
ZNF595	2				ZNF595		ZNF595			
ZNF335	2	ZNF335							ZNF335	
ZNF236	2			ZNF236						ZNF236
ZNF225	2			ZNF225			ZNF225			
ZFYVC26	2			ZFYVC26						ZFYVC26
ZCCHC4	2			ZCCHC4						ZCCHC4
VPS37C	2			VPS37C		VPS37C				
VIT	2			VIT						VIT
VAV2	2		VAV2							VAV2
USP2	2		USP2	USP2						
TXNDC5	2		TXNDC5							TXNDC5
TTLL8	2			TTLL8		TTLL8				
TSPAN16	2			TSPAN16				TSPAN16		
TRPM5	2			TRPM5				TRPM5		
TRAF2	2	TRAF2				TRAF2				
TRA2B	2			TRA2B			TRA2B			
TPP1	2				TPP1	TPP1				
TOP2B	2		TOP2B		TOP2B					
TNFRSF1B	2			TNFRSF1B			TNFRSF1B			

After removing the NC group data, part of the mutant genes in the nIHH group (from high to low frequency)

**Table 3:** The presence of IHH pathogenic gene has been determined in group nIHH

	nIHH1	nIHH2	nIHH3	nIHH4	nIHH5	nIHH6	nIHH7	nIHH8	nIHH9
CHD7		⚪		⚪					
ANOS1				⚪					
FGFR1			⚪						
SEMA7A			⚪						
OTUD4		⚪							
AMH									⚪
KLB				⚪					
GATA2							⚪		
POLR3B							⚪		

After excluding the NC group data, the number of mutant genes related to IHH pathogenesis identified in previous studies included in the mutant genes in the nIHH group

Subsequently, we performed an analysis based on the ACMG criteria and summarised the results. There were 41 variants identified as pathogenic or likely pathogenic based on the ACMG criteria. Variants in *ADAMTS6* and *COL12A1* were identified in two patients. The *ADAMTS6* variant identified in patients nIHH7 and nIHH8 was 64747447A > C. Only the *FGFR1* variant in nIHH patient 3 was a previously identified IHH pathogenic variant (Table 4).

**Table 4:** ACMG analyses of nIHH

	nIHH1	nIHH2	nIHH3	nIHH4	nIHH5	nIHH6	nIHH7	nIHH8	nIHH9
***P***	PDZD4	WDPCP	FGFR1	NCAPG2	CCNO	CFH	CLASP1	ADAMTS6	MRC2
	PBRM1	EYA1		TANC2	ABCB4	IL36RN	ATP13A3	MC4R	ZNF236
	SRP54					UBR3	ADAMTS6		
							CACNB1		
							ELL		
***LP***	DSP		PLEKHO1	SLC4A11	RYR2	SECISBP2	GLYCTK	EIF2B5	PYCR2
	BRF1		**COL12A1**	KDM6B	KLHL3		**COL12A1**	PGAM2	CPA6
			FIG4		PRKCH		PLG		NARS2
			**TMPRSS3**						LENG8
			**TMPRSS3**						
			NCF4						

*P* pathogenic, *LP* likely pathogenic. Bold mutation gene appears twice or more

An analysis of variants that were repeated in the nIHH group showed that variants in *THSD1*, *SMPD1*, *SCL16A3*, and *KDM2B* were each identified in three patients. These genes showed the highest repetition rate in the nIHH group, but no variants in these genes were found in any other group.

In the pathway enrichment analysis of the nIHH group, after excluding variants detected in the NC group, each nIHH patient was found to have 12–41 enriched pathways, and 12 pathways were enriched in more than two patients. Of these, the cell division pathway (GO: 0051301) was enriched in three patients: nIHH3, nIHH4, and nIHH9 (Table 5).

**Table 5:** Enrichment pathways of nIHH

	Num	nIHH1	nIHH2	nIHH3	nIHH4	nIHH5	nIHH6	nIHH7	nIHH8	nIHH9
*GO:0051301: cell division*	3			⚪	⚪					⚪
*GO:0090066: regulation of anatomical structure size*	2	⚪				⚪				
*M5880: NABA ECM AFFILIATED*	2	⚪						⚪		
*hsa04142: Lysosome*	2			⚪		⚪				
*GO:0009410: response to xenobiotic stimulus*	2						⚪	⚪		
*R-HSA-2672351: Stimuli-sensing channels*	2							⚪	⚪	
*GO:0009582: detection of abiotic stimulus*	2		⚪	⚪						
*GO:0030111: regulation of Wnt signalling pathway*	2		⚪					⚪		
*GO:0035176: social behaviour*	2				⚪	⚪				
*GO:0050905: neuromuscular process*	2				⚪	⚪				
*R-HSA-6811442: Intra-Golgi and retrograde Golgi-to-ER traffic*	2				⚪				⚪	
*GO:0045197: establishment or maintenance of epithelial cell apical/basal polarity*	2					⚪		⚪		

After screening of enrichment pathways, some enrichment pathways included in the nIHH group (from high to low frequency)

### Co-analysis of the Three Patient Groups

Nine nIHH patients, 19 VC patients, and five SN patients shared 116 variants, with 28 variant-harbouring genes detected in five or more patients. *PRRC2A* was the gene most involved, with variants in this gene detected in one SN patient (SN3) and eight nIHH and VC patients. *AKAP13* variants were detected in eight patients; *MICAL* variants were detected in seven patients; and *PBRM1*, *RECQL5*, *DOCK8*, *DCAF13*, and *SLC26A4* variants were detected in six patients. *RECQL5* variants were detected in six patients, five of whom had had splice-site variants at locus 73,626,919 (Table 6). The genetic variants detected in each group are presented in Supplementary Materials.

**Table 6:** Overview of common mutations in three groups except NC

	nIHH1	nIHH2	nIHH3	nIHH4	nIHH5	nIHH6	nIHH7	nIHH8	nIHH9	SN1	SN2	SN3	SN4	SN5
PRRC2A			PRRC2A			PRRC2A	PRRC2A		PRRC2A			PRRC2A		
AKAP13					AKAP13						AKAP13		AKAP13	
MICAL3				MICAL3		MICAL3					MICAL3			
PBRM1	PBRM1						PBRM1	PBRM1			PBRM1			
RECQL5	RECQL5		RECQL5										RECQL5*2	
SLC26A4				SLC26A4							SLC26A4	SLC26A4		
DOCK8					DOCK8						DOCK8			
DCAF13				DCAF13									DCAF13	DCAF13
HGFAC					HGFAC	HGFAC	HGFAC				HGFAC			
GPR158	GPR158		GPR158			GPR158					GPR158			
RYR2				RYR2	RYR2						RYR2			
PCSK6			PCSK6					PCSK6				PCSK6		
NCKAP5				NCKAP5			NCKAP5				NCKAP5*2	NCKAP5		
ITGB5			ITGB5		ITGB5								ITGB5	
FOXN1						FOXN1		FOXN1					FOXN1	FOXN1
FLII	FLII				FLII					FLII				FLII
ENKUR		ENKUR						ENKUR				ENKUR		
USP40								USP40		USP40		USP40		
PPFIBP2					PPFIBP2					PPFIBP2				
PCDH15							PCDH15						PCDH15*2	
NEK4							NEK4				NEK4			NEK4
NBEAL1							NBEAL1			NBEAL1				
MUC17				MUC17*2									MUC17	
DCT				DCT								DCT		
CUBN								CUBN				CUBN*2	CUBN	
CHD9									CHD9					CHD9
C1orf167						C1orf167					C1orf167	C1orf167*2		
ANKRD11		ANKRD11									ANKRD11			ANKRD11

	VC1	VC2	VC3	VC4	VC5	VC6	VC7	VC8	VC9	VC10	VC11	VC12	VC13	VC14
PRRC2A					PRRC2A			PRRC2A			PRRC2A*2			
AKAP13			AKAP13*2	AKAP13	AKAP13	AKAP13		AKAP13						
MICAL3	MICAL3	MICAL3*2												MICAL3
PBRM1													PBRM1	PBRM1
RECQL5	RECQL5					RECQL5								
SLC26A4								SLC26A4						SLC26A4
DOCK8						DOCK8	DOCK8	DOCK8				DOCK8		
DCAF13										DCAF13				DCAF13
HGFAC														
GPR158														
RYR2														
PCSK6		PCSK6												
NCKAP5		NCKAP5												
ITGB5		ITGB5							ITGB5					
FOXN1					FOXN1									
FLII														
ENKUR						ENKUR								
USP40									USP40					USP40
PPFIBP2			PPFIBP2			PPFIBP2				PPFIBP2				
PCDH15			PCDH15		PCDH15									
NEK4												NEK4	NEK4	
NBEAL1			NBEAL1		NBEAL1									
MUC17										MUC17	MUC17			MUC17
DCT		DCT			DCT									
CUBN			CUBN											
CHD9	CHD9	CHD9											CHD9	
C1orf167								C1orf167						
ANKRD11					ANKRD11						ANKRD11			

	VC15	VC16	VC17	VC18	VC19	Num (nIHH)	Num (SN)	Num (VC)	Num (total)					
PRRC2A					PRRC2A	4	1	4	9					
AKAP13						1	2	5	8					
MICAL3			MICAL3			2	1	4	7					
PBRM1						3	1	2	6					
RECQL5			RECQL5			2	1	3	6					
SLC26A4		SLC26A4				1	2	3	6					
DOCK8						1	1	4	6					
DCAF13	DCAF13					1	2	3	6					
HGFAC			HGFAC			3	1	1	5					
GPR158	GPR158					3	1	1	5					
RYR2			RYR2	RYR2		2	1	2	5					
PCSK6		PCSK6				2	1	2	5					
NCKAP5						2	2	1	5					
ITGB5						2	1	2	5					
FOXN1						2	2	1	5					
FLII				FLII		2	2	1	5					
ENKUR		ENKUR				2	1	2	5					
USP40						1	2	2	5					
PPFIBP2						1	1	3	5					
PCDH15			PCDH15			1	1	3	5					
NEK4						1	2	2	5					
NBEAL1	NBEAL1					1	1	3	5					
MUC17						1	1	3	5					
DCT			DCT			1	1	3	5					
CUBN			CUBN			1	2	2	5					
CHD9						1	1	3	5					
C1orf167					C1orf167	1	2	2	5					
ANKRD11						1	2	2	5					

Some gene mutations common to nIHH, SN, and VC groups (from high to low frequency)

In the enrichment pathway analysis, only nine pathways were identified in all three groups of patients. “M5880: NABA ECM AFFILIATED”, “R-HSA-9675108: nervous system development”, and “GO:0090066: regulation of anatomical structure size” were identified in five patients; “R-HSA-9716542: signalling by Rho GTPases”, “Miro GTPases”, and “RHOBTB3” were identified in four patients; and the other pathways were identified in three patients (Table 7). Details of enrichment analysis in each group are presented in Supplementary Materials.

**Table 7:** Overview of common enrichment pathways in three groups except NC

	Num	nIHH1	nIHH2	nIHH3	nIHH4	nIHH5	nIHH6	nIHH7	nIHH8	nIHH9	SN1	SN2
*M5880: NABA ECM AFFILIATED*	5	⚪						⚪			⚪	
*R-HSA-9675108: nervous system development*	5							⚪				
*GO:0090066: regulation of anatomical structure size*	5	⚪				⚪						
*R-HSA-9716542: signaling by Rho GTPases, Miro GTPases, and RHOBTB3*	4						⚪				⚪	
*GO:0060537: muscle tissue development*	3							⚪			⚪	
*R-HSA-111465: apoptotic cleavage of cellular proteins*	3						⚪					⚪
*GO:0006913: nucleocytoplasmic transport*	3							⚪				
*hsa00310: lysine degradation*	3		⚪									
*GO:0006974: cellular response to DNA damage stimulus*	3						⚪					

	SN3	SN4	SN5	VC1	VC2	VC3	VC4	VC5	VC6	VC7	VC8	VC9
*M5880: NABA ECM AFFILIATED*							⚪					
*R-HSA-9675108: nervous system development*		⚪								⚪		⚪
*GO:0090066: regulation of anatomical structure size*		⚪										
*R-HSA-9716542: signaling by Rho GTPases, Miro GTPases, and RHOBTB3*												
*GO:0060537: muscle tissue development*												
*R-HSA-111465: apoptotic cleavage of cellular proteins*												
*GO:0006913: nucleocytoplasmic transport*	⚪								⚪			
*hsa00310: lysine degradation*		⚪										
*GO:0006974: cellular response to DNA damage stimulus*		⚪										

	VC10	VC11	VC12	VC13	VC14	VC15	VC16	VC17	VC18	VC19		
*M5880: NABA ECM AFFILIATED*					⚪							
*R-HSA-9675108: nervous system development*										⚪		
*GO:0090066: regulation of anatomical structure size*			⚪				⚪					
*R-HSA-9716542: signaling by Rho GTPases, Miro GTPases, and RHOBTB3*								⚪				
*GO:0060537: muscle tissue development*				⚪								
*R-HSA-111465: apoptotic cleavage of cellular proteins*	⚪											
*GO:0006913: nucleocytoplasmic transport*												
*hsa00310: lysine degradation*		⚪										
*GO:0006974: cellular response to DNA damage stimulus*			⚪									

Some enrichment pathways shared by nIHH, SN, and VC groups (from high to low frequency)

### Comparison of Different Variant Sites in the Same Gene

We screened 17 variant-harbouring genes with the highest frequency among patients in the nIHH group (*TTN*, *IST1*, *NEFH*, *CCDC177*, *TRIP10*, *FAM174B*, *USH2A*, *PCLO*, *CASQ2*, *BPTF*, *MUC19*, *ALMS1*, *PLEC*, *NT5DC4*, *MUC17*, *RIC8A*, and *OBSCN*) and determined the status of these 17 genes in all patients (Table 8). Mutations in these genes occur in the exon region of the corresponding gene and alter the amino acid sequence of the expressed protein, thereby hindering the normal expression of the corresponding gene; the detailed mutation situation is shown in the table (Supplementary Materials). And some of the mutations were positively confirmed by Sanger sequencing on the corresponding patients. Variants in two genes, *TTN* and *IST1*, were detected in 83% (38/46) and 98% (45/46) of patients, respectively. Variants in *NEFH*, *CCDC177*, *TRIP10*, *FAM174B*, and *USH2A* were detected in more than 50% of patients, and the selected 17 genes accounted for more than 25% of the variants detected.

**Table 8:** Overview of common mutations in four groups

Gene	Proportion(nIHH)	Proportion (SN)	Proportion (VC)	Proportion (NC)	Proportion (total)
TTN	9/9	4/5	15/19	10/13	38/46
IST1	9/9	4/5	19/19	13/13	45/46
NEFH	8/9	2/5	9/19	7/13	26/46
CCDC177	8/9	0/5	4/19	4/13	16/46
TRIP10	6/9	3/5	3/19	0/13	12/46
FAM174B	6/9	2/5	10/19	7/13	19/46
USH2A	6/9	0/5	6/19	4/13	16/46
PCLO	5/9	0/5	2/19	0/13	7/46
CASQ2	5/9	1/5	6/19	0/13	12/46
BPTF	4/9	5/5	10/19	7/13	26/46
MUC19	4/9	3/5	5/19	2/13	14/46
ALMS1	4/9	2/5	4/19	3/13	13/46
PLEC	4/9	3/5	9/19	2/13	18/46
NT5DC4	4/9	2/5	9/19	5/13	20/46
MUC16	3/9	4/5	8/19	8/13	23/46
RIC8A	3/9	2/5	6/19	5/13	19/46
OBSCN	2/9	3/5	7/19	4/13	16/46

The sequence of mutant genes is from more to less according to the number of repeats in group nIHH

**Fig. 3:**
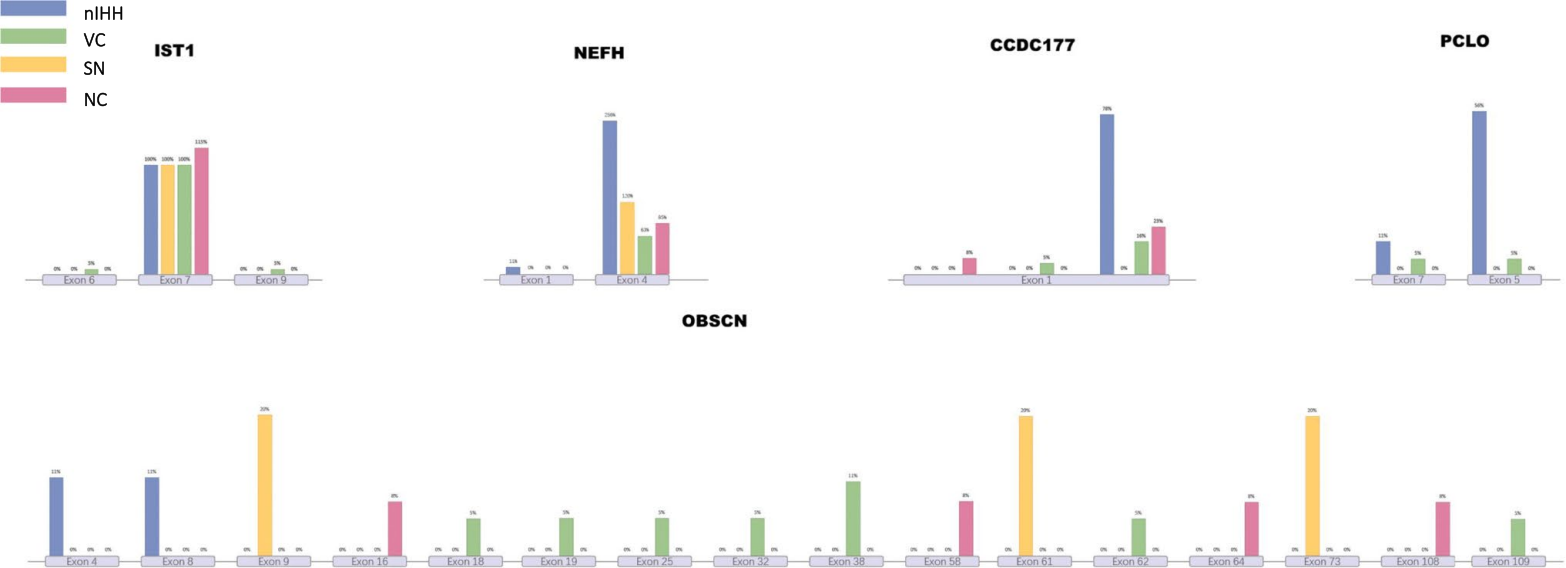
Specific exon positions of different groups in the same mutant gene. Each gene corresponds to the specific exon of the gene in which the mutations of different groups of patients are located. The percentage shows that more than 100% of the mutations are multiple mutations of the gene in the same patient

For specific variant sites and exons, we present the details of some genes for each group (Fig. 3). Of the *NEFH* variants detected, seven nIHH patients had variants in exon 4; one nIHH patient had a variant in exon 1; and the remaining two SN patients, nine VC patients, and seven NC patients had variants in exon 4. In addition, 21 variants in the *NEFH* gene were detected in seven nIHH patients, whereas only six *NEFH* variants were detected in the SN group, 12 in the VC group, and 11 in the NC group.

Seven nIHH patients had a *CCDC177* variant located in exon 1, and all of these were at locus 70,039,793. There were three and four *CCDC177* variants detected in the VC and NC groups, respectively. These variants were all located in exon 1 but were not all at the same site as the variant detected in the nIHH patients. *PCLO* variants were almost exclusively found in nIHH patients, and five of the six *PCLO* variants were in exon 5.

For *OBSCN*, two variants were detected in the nIHH group, three were detected in the SN group, seven were detected in the VC group, and four were detected in the NC group. The *OBSCN* variants were scattered across 16 exons. There were 19 *TTN* variants in the nIHH group, seven in the SN group, 33 in the VC group, and 23 in the NC group, scattered across 47 exons. Exon 276 was the most frequent site for *TTN* variants, with four in the nIHH group, one in the SN group, two in the VC group, and four in the NC group. The details of the variant sites are provided in the Supplementary Materials.

## Discussion

We selected nIHH, VC, and SN patients and healthy individuals as the experimental subjects. By excluding genetic variants detected in healthy individuals and comparing the variants detected in the three experimental groups, we aimed to identify the specific genetic variants associated with nIHH.

First, we compared the nIHH data with previously identified IHH pathogenic genes. Nine variant-harbouring genes were found in nine patients, with only a *CHD7* mutation detected in two patients. This is consistent with previous reports and knowledge of nIHH from the literature, indicating that there is obvious genetic heterogeneity among IHH patients, with no variants in single genes or gene classes cluster in IHH patients on a large scale. It is worth noting that previous studies of *CHD7* have focused on KS, CHARGE syndrome, and myocardial function, whereas our two patients with *CHD7* variants did not have olfactory dysfunction or other systemic diseases [1, 25–27]. Moreover, in the analysis of the nIHH group based on the ACMG criteria, only variants in the *FGFR1* gene were identified as pathogenic. *ADAMTS6* and *COL12A1* variants were detected in two nIHH patients. The site and type of variant were identical in the two patients with *ADAMTS6* variants. This is the first report of *ADAMTS6* variants associated with male-infertility-related diseases. Only one study has reported an association between this gene and developmental delay, suggesting that *ADAMTS6* variants may affect a certain process during growth and development [28]. Of the genes with intragroup duplication, variants in *SMPD1* have previously been shown to increase α-synuclein levels and impair acid sphingomyelinase trafficking to lysosomes to induce the development of Alzheimer’s disease [29]. Niemann–Pick disease has also been shown to be related to variants in *SMPD1*, and type A Niemann–Pick disease manifests with central nervous system abnormalities [30]. Variants in the *SMPD1* gene may affect the regulation of the gonad–pituitary axis from the central nervous system and thus affect the development of nIHH. It has also been found that KDM2B inhibits the expression of somatic genes and thus inhibits somatic differentiation during the specification of human primordial germ-cell-like cells, which may be one of the reasons for the azoospermia phenotype in nIHH patients [31].

The results of our pathway analysis showed that only one pathway, cell: division, was enriched in three patients, and 11 pathways were enriched in two patients. None of the 12 enriched pathways has previously been reported to be related to the pathogenesis of nIHH, nor have these pathways been associated with azoospermia. The current hypothesis for the basic aetiology of nIHH, namely, that it originates from damage to GnRH neurons and impaired GnRH synthesis and release, does not appear to be supported by our enrichment analysis results. Our enrichment analysis of a single group of genes with variants in nIHH also showed strong heterogeneity. We did not find any meaningful enrichment of biological pathways involved in GnRH function, that is, the biological function of the hypothalamus or pituitary gland. However, we found many other functional abnormalities, such as cell division, which may be related to spermatogenesis. As mentioned above, variants in *KDM2B* cause abnormalities in the peripheral reproductive system by affecting the division and proliferation of germ cells. However, our biological function pathway analyses showed great heterogeneity, and therefore, we cannot draw a unified simple conclusion about the pathogenic mechanism of nIHH gene variants. However, our findings show that the main pathogenic basis of nIHH may be in the peripheral, rather than the central and nervous system, and that abnormalities of GnRH function caused by genetic variants is not the only pathogenic mechanism of nIHH.

Many genetic variants were common between nIHH, VC, and SN, but many of them have not been reported to be associated with nIHH or other male infertility disorders. However, some of these variants have been found to be associated with male infertility. The methylation level of *PRRC2A* has been shown to be significantly correlated with sperm number and sperm motility. Moreover, in a study of NOA in the Han Chinese population, *PRRC2A* variants were found to lead to abnormal spermatogenesis [32]. A recent study of IHH also suggested that PLXNB1 may induce IHH by affecting changes to GnRH neurons [33]. In addition, some genes have also been found to affect the function of the nervous system. For example, *COL11A2* is related to genetic hearing loss and deafness, which is also consistent with some of the phenotypes in our nIHH patients [34, 35]. In the enrichment analysis of three sets of intersecting data, three pathways were enriched in five patients. Of these, the “regulation of the anatomical structure size” pathway, as its name implies, is a collection of genes regulating cellular shape and structure. Most of the genes in this pathway are actin-related genes. Variants in these genes may result in abnormal sperm structure or developmental disruption to a certain extent. Rho-GTPase-related pathways were also enriched in four patients, and these pathways are important in the formation of the actin cytoskeleton, which supports the abovementioned view from another perspective [36]. These results show that patients with IHH, SN, and even some with VC (severe sperm abnormalities) may have common pathogenic genetic variants or enrichment pathways. Moreover, the effects caused by these variants can only be located in the peripheral, rather than the central and nervous system because there were no central nervous system-related symptoms in the SN or VC groups in our study. Although these variants (such as those in *PRRC2A*) or pathways are not the only cause of IHH, they may be part of the genetic cause and pathogenic mechanism in some IHH patients.

In the initial analysis, many genetic variants shared by the experimental groups and the control group were filtered out so that we only focused on variants that differed in number between the experimental groups and the control group. However, in a subsequent analysis, we found that some variants were frequent in both the nIHH and control groups. These genes have been characterised in the 1000 Genomes Project and found to have a low frequency in the population. We performed an in-depth analysis of the specific exons and sites containing variants for these high consensus genes. In general, these genes could be divided into three categories. The first category included *IST1* and *NT5DC4*. These variants were all located in the same exon, and there was no significant statistical difference in their frequency between groups. *IST1* encodes a protein with microtubule-interacting-and-trafficking-interacting motifs that interact with components of endosomal sorting complexes required for transport (ESCRT) [37–39]. The IST1 protein regulates the ESCRT-III complex to drive membrane deformation and fission. The role of *NT5DC4* is unclear. As the variants detected in this gene occur at a high frequency in the Han Chinese population, they are likely to have no clinical significance. The second category included *TTN* and *OBSCN*. Variants in these gene were quite scattered, both in the number of groups and in the exons or specific sites where the variants were located. The third category included *CCDC177*, *NEFH*, and *PCLO*. The number of variants in these genes was significantly greater in the nIHH group than the other groups. The protein encoded by the *PCLO* gene is part of the presynaptic cytoskeleton matrix, which is involved in the establishment of active synaptic regions and synaptic vesicle trafficking. Some studies have shown that PCLO may be related to human affective disorder, depression, and type 2 diabetes [40, 41]. *NEFH* encodes a heavy neurofilament protein, which is associated with neuronal damage [42]. Variants in *NEFH* may be the cause of Charcot–Marie–Tooth neuropathy. However, these variants have not been reported to be related to azoospermia or other related diseases. Our data showed an abnormally high frequency of these variants in IHH, suggesting that they may be related to the pathogenic mechanism of the disease, but this requires further validation and functional research.

The new analysis strategy used in this study provides a new perspective from which to explore the true pathogenic mechanism of nIHH. We conclude that the *NEFH*, *CCDC177*, and *PCLO* genes show an abnormally high variant frequency in nIHH and the pathways GO:0051301: cell division and GO:0090066: regulation of anatomical structure size may be the key difference between nIHH, other types of NOA, and VC. Our results suggest that the pathogenic mechanism of nIHH is not entirely based on GnRH dysfunction. The pathological mechanism of nIHH is not limited to the effects of GnRH on the central nervous system, and other heterogeneous pathogenic genetic variants affecting peripheral organs may also be involved.

### Supplementary Information

Below is the link to the electronic supplementary material.

Supplementary file1 (XLSX 248 KB)

## Data Availability

All available published data on this study have been included in the main document, tables, figures, and supplementary materials.
